# Menopausal transitional and postmenopausal women’s voices: “what influences their adherence to self-management”: a systematic review and meta-synthesis of qualitative studies

**DOI:** 10.3389/fpubh.2025.1653806

**Published:** 2025-10-29

**Authors:** Yan Jiang, Minfang Tao, Cuiqin Huang, Qunfeng Lu

**Affiliations:** Shanghai Sixth People's Hospital Affliated to Shanghai Jiaotong University School of Medicine, Shanghai, China

**Keywords:** perimenopause, climacteric, self-management, qualitative research, systematic review

## Abstract

**Background:**

Self-management plays a vital role in preventing the occurrence of severe menopausal symptoms and hazards. There has been a lack of systematic reviews exploring the influencing factors and challenges faced by menopausal transitional and postmenopausal women regarding their self-management experiences and perceptions.

**Objective:**

This study aimed to synthesize the self-management experiences, motivations, and challenges of menopausal transitional and postmenopausal women, to inform the design and development of self-management programs. The results were analyzed according to the capability, opportunity, and motivation model of behavior (COM-B) model, and suggestions for improvement were subsequently put forward.

**Methods:**

A meta-aggregation method was used to synthesize qualitative studies. Ten databases were searched for studies published up to 3 April 2025. Two researchers assessed the quality and risk of bias and extracted data from the included studies independently. A thematic synthesis approach was used to analyze the key findings using the COM-B model.

**Results:**

A total of 23 primary studies containing 808 participants were included. Six themes with fifteen sub-themes were recognized after reading and coding of the articles: Poor cognition (misconceptions about menopause and treatment, insufficient knowledge, lack of active health literacy, and lack of empowerment); physical restriction (medical conditions and fatigue); restricted environment (limited resources and restrictions on amenities); impact of interpersonal circle (the influence of family support and understanding, social belonging, and lack of useful advice from experts); planning and adherence (setting a solid plan and the psycho-immune system); and expectations for keeping health (perceived health benefits and health crisis concern). Analysis of the findings according to the COM-B model revealed that poor cognition, physical restriction, and restricted environment are the challenges faced by menopausal transitional and postmenopausal women. Expectations for keeping healthy serve as the motivation for self-management behaviors. Impact of the interpersonal circle, planning, and adherence are the important influencing factors in maintaining self-management behavior in menopausal transitional and postmenopausal women.

**Conclusion:**

This study shows that knowledge, empowerment, and family and social support are key motivators for self-management among perimenopausal and postmenopausal women. We believe that addressing perimenopausal and postmenopausal women’s knowledge needs, improving their knowledge, empowering the skills of healthcare providers, and reinforcing the supportive environment for self-management behaviors, including physical activity, access to community health services, and a supportive interpersonal environment, are effective interventions to promote self-management.

**Systematic review registration:**

The protocol for this study has been registered with PROSPERO(Registration Number: CRD420251025120). https://www.crd.york.ac.uk/PROSPERO/view/CRD420251025120.

## 1 Introduction

Menopause is the cessation of menstruation, usually confirmed after 12 consecutive months of amenorrhea, and the World Health Organization (WHO) estimated that almost 1 billion women worldwide will be menopausal, transitional, and postmenopausal by 2050 (1). The results of a meta-analysis with 82,340 Chinese women aged 40 to 65 years old showed that the incidence of perimenopausal syndrome was up to 61.0% (2).

The menopausal transition is a distinct phase that can last between 2 and 8 years before menopause (3). During the menopausal transition and postmenopausal stage, there will be a range of physical and psychological symptoms due to declining estrogen levels and fluctuations in the menstrual cycle, ultimately resulting in a reduced quality of life (3). Furthermore, there is an increased incidence of cardiovascular disease and osteoporosis after menopause (4). In China, the word “climacteric” is sometimes used as a derogatory term to indicate a person’s temper and capriciousness, carrying a negative connotation. Menopausal women are regarded as “mentally ill” or “abnormal people,” contributing to the taboo surrounding this topic (5). Not only does menopause have an impact on individual well-being, but it also has a significant impact on women in the workplace. Studies have shown that more than 65% of women suffer from menopausal symptoms, which are often exacerbated by a lack of a supportive work environment (6–8). Thus, menopause is not only a health problem but also a social and work problem. The concept of health menopause has been focused on by studies and was defined as a dynamic state following permanent loss of ovarian function characterized by self-perceived satisfactory physical, psychological, and social functioning, including disease and disability, enabling women to achieve needed resilience and self-management (9). The article published by The Lancet also mentioned the use of the Empower model to support self-managed health (10). Therefore, self-management is essential for achieving a healthy menopause.

Self-management means that patients maintain and promote their own health by managing their own behavior to reduce the impact of the disease on physical, psychological, and mental health (11), emphasizing the patient’s ability and behavior to manage symptoms (12, 13). Active self-management was defined as taking action, such as taking non-HRT medications, using alternative therapies, and making lifestyle changes to treat the symptoms (14).

Self-management during the menopausal transition provides the opportunity to achieve the goal of supporting women through the menopausal transition to manage bothersome symptoms and promote health in old age. Menopausal transition and postmenopausal lifestyle habits are associated with an increased risk of chronic diseases. It is possible to improve this risk by following a healthy lifestyle, including regular exercise and a balanced diet, to achieve a healthy menopause (15). Physical activity has significant positive effects on cardiometabolic, physical, and mental health in middle-aged women, especially when performed during the menopausal transition and postmenopausal years (16). Self-management is mainly used in health promotion and health education related to patients with chronic diseases, and it has become a research hotspot of health-promoting behavior (13). However, a systematic review of self-management strategies for menopausal symptoms revealed inadequate awareness and use of culture-based remedies, which can have deleterious effects on menopausal acceptance and symptom management (17). Thus, we need to explore the experience and perception of self-management for menopausal transitional and postmenopausal women.

In quantitative studies, questionnaires or scales can be used to analyze the influencing factors of self-management ability in perimenopausal and postmenopausal women (18, 19); however, these studies cannot explain the experience and feelings of perimenopausal women in self-management and cannot provide a reasonable explanation for perimenopausal women to adhere to regular self-management behavior. However, qualitative studies make up for those shortcomings. At present, there have been relevant qualitative studies exploring the experiences of self-management in perimenopausal women, but there is still a lack of integration of these research results to understand the phenomenon comprehensively and deeply, so as to make the results more comprehensive and credible. Therefore, the aim of this study was to summarize the self-management experiences, motivations, and challenges among menopausal transitional and postmenopausal women and then fill this gap.

The COM-B model is a theoretical framework that identifies three critical factors influencing human behavior: capability, opportunity, and motivation. It serves as a guide for proposing and applying targeted nursing interventions in implementation science (20).

In conclusion, the objectives of this study were (1) to conduct a systematic and comprehensive qualitative study on the experiences, motivations, and needs of self-management among perimenopausal and postmenopausal women; (2) the COM-B model was used to analyze the results of this research, understand the influencing factors, and propose the corresponding intervention measures.

## 2 Methods

### 2.1 Aims and design

This study aimed to systematically synthesize qualitative evidence on self-management experiences and influencing factors and challenges in perimenopausal and postmenopausal women. The review protocol has been registered on PROSPERO (number CRD420251025120), and this review was conducted following the Joanna Briggs Institute (JBI) Methodology Manual (21). The reporting of our study follows the Enhancing Transparency in Reporting the Synthesis of Qualitative Research (ENTREQ) statement (22).

### 2.2 Search strategy

Multiple databases were searched: PubMed, Embase, Medline (via Ovid), Web of Science Core Collection, CINAHL (via EBSCO), SCOPUS, PsycInfo (via EBSCO), China National Knowledge infrastructure (CNKI), China Online Journals (COJ), China Science and Technology Journal database (VIP), using a combination of subject terms and free words. A three-step search strategy was adopted according to the JBI Methodology Guide (21). First, an initial search in the PubMed database was conducted before registration to decide the feasibility of this review and analyze the vocabulary and subject terms in the titles and abstracts. The second step involved using the constructed computer search in the target databases. Third, relevant references for the retrieved article were searched to prevent omission of other relevant studies. The research strategy focused on articles published up to 3 April 2025. All the searched details are available in Supplementary file 1.

### 2.3 Inclusion and exclusion criteria

The inclusion criteria and exclusion criteria are shown in Table 1.

**Table 1:** Article inclusion and exclusion criteria.

Study aspect	Inclusion criteria	Exclusion criteria
Design	Qualitative methodology or mixed methods	Mixed-method studies with predominantly quantitative results
Aim/focus	The real experience, motivation, and needs of self-management of menopausal transitional and postmenopausal women	Only focused on the experience of menopause without mentioning the motivation or needs of self-management
Article type	Peer-reviewed journal articles	-
Language	English or Chinese	-
Participants	Menopausal transitional and postmenopausal women	People who have been diagnosed with other severe diseases (i.e., bilateral oophorectomy, hysterectomy), which result in menopause
Others	-	Unavailability of the full text; repeated or overlapping publications.

### 2.4 Study screening and selection

The research process is shown using the Preferred Reporting Items for Systematic Reviews and Meta-Analyses (PRISMA) flow diagram. All references were imported into EndNote 20 software, and duplicates were removed. Two researchers (JY and HC) trained in systematic review methods and screened all of the studies independently. Any disagreements were resolved through consultation between the two investigators or further discussion with a third investigator (TM).

### 2.5 Quality assessment

The JBI Quality Evaluation Criteria for Qualitative Research (21) was used to evaluate the quality of the included studies by two researchers (JY and HC) independently. The evaluation criteria consist of 10 questions with “yes,” “no,” “unclear,” or “not applicable” answers. Included studies with no or unclear answers to over five questions were identified as low quality and excluded from this review. Any disagreements were resolved through discussion between the two researchers or through further discussion with a third researcher (TM).

### 2.6 Data extraction

Data extraction from included articles was conducted independently by two researchers (JY and HC) according to the JBI Qualitative Assessment and Review Instrument data extraction tool (21) and a crosscheck by a third researcher (TM), including author, year of publication, country, study design, data collection method, methodology for data analysis, participants, aims, and main findings.

### 2.7 Data synthesis

Data were thematically synthesized using Thomas and Harden’s three-stage framework (23). First, familiarization with data was achieved through line-by-line reading, rereading, and coding of the results sections from the primary studies related to self-management, carried out independently by two researchers (JY and HC). In Stage 2, “free codes” were grouped into related areas to form “descriptive” themes aligned with the research questions. The final stage involved the development of “analytical” themes. NVivo11 software was used for meaning, understanding, and content comparison. Any disagreements were resolved through discussion between the two researchers or through further discussion with a third researcher (TM).

### 2.8 Researcher reflexivity and validity

Many of the authors had experience in menopausal women’s education and instruction for self-management or researching self-management, or symptom management interventions or programs in health settings or communities. These previous experiences suggested that organizational and staff attitudinal barriers were particularly influential when compared to influencing factors related to menopausal women themselves. The impact of these preconceptions was minimized through regular reflective discussions with the broader researcher team, which included a background in implementation science and no previous experience in menopause clinics or other relevant health settings. This interdisciplinary discussion process helped ensure that researchers were exposed to alternative explanations and increased validity.

## 3 Results

### 3.1 Literature search

**Figure 1:**
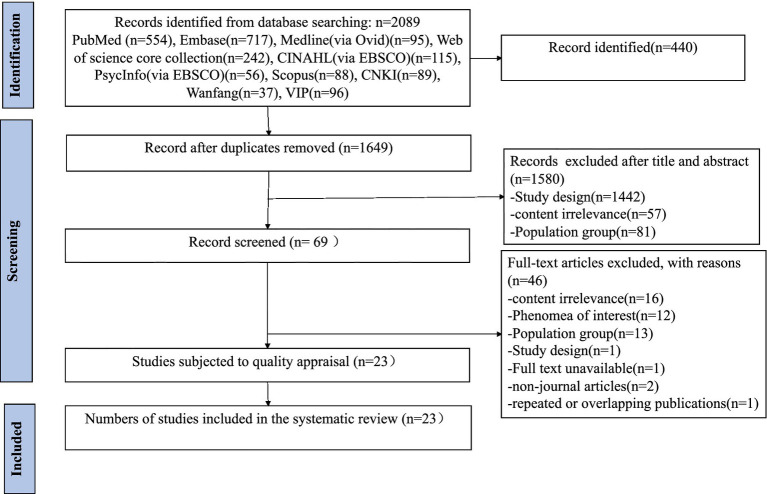
Flowchart of the research process.

As shown in Figure 1, database searches yielded 2,089 results, with 1,649 records remaining after the removal of 440 duplicates. Finally, after removing articles according to the inclusion and exclusion criteria, 23 studies were included.

### 3.2 Risk of bias in studies

Table 2 shows the quality assessment results of the included studies that were independently evaluated by two researchers (JY and HC). All of the included studies demonstrated good methodological quality, with scores ranging from 70 to 100%.

**Table 2:** Results of the quality assessment.

Study	Criterion										Ratio
	C1	C2	C3	C4	C5	C6	C7	C8	C9	C10	
Chiang et al. (2009) (28)	Y	Y	Y	Y	Y	U	U	Y	N	Y	70%
Jeng et al. (2004) (29)	Y	Y	Y	Y	Y	N	N	Y	Y	Y	80%
Im et al. (2011) (24)	Y	Y	Y	Y	Y	U	U	Y	Y	Y	80%
Xiong et al. (2012) (31)	U	Y	Y	Y	Y	N	N	Y	N	Y	80%
Doubova (2012) (39)	Y	Y	Y	Y	Y	N	N	Y	Y	Y	80%
Odiari et al. (2012) (14)	Y	Y	Y	Y	Y	N	N	Y	N	Y	80%
Lee et al. (2014) (30)	Y	Y	Y	Y	Y	Y	Y	Y	Y	Y	100%
Mackey et al. (2014) (40)	Y	Y	Y	Y	Y	N	N	Y	Y	Y	80%
McArthur et al. (2014) (41)	Y	Y	Y	Y	Y	N	N	Y	Y	Y	80%
Hardy et al. (2017) (42)	Y	Y	Y	Y	Y	N	N	Y	Y	Y	80%
Bahri et al. (2017) (37)	Y	Y	Y	Y	Y	Y	N	Y	Y	Y	90%
Herbert et al. (2020) (43)	Y	Y	Y	Y	Y	N	N	Y	Y	Y	80%
Kim et al. (2020) (44)	Y	Y	Y	Y	Y	N	Y	Y	Y	Y	90%
Pimenta et al. (2020) (33)	Y	Y	Y	Y	Y	N	N	Y	U	Y	80%
Asad et al. (2021) (45)	Y	Y	Y	Y	Y	N	U	Y	Y	Y	80%
Ilankoon et al. (2021) (35)	Y	Y	Y	Y	Y	Y	Y	Y	Y	Y	100%
Berin et al. (2022) (36)	Y	Y	Y	Y	Y	N	N	Y	Y	Y	80%
Kracht et al. (2022) (25)	Y	Y	Y	Y	Y	N	N	Y	Y	Y	80%
Cortes et al. (2024) (26)	Y	Y	Y	Y	Y	N	N	Y	Y	Y	80%
Zhang et al. (2024) (32)	Y	Y	Y	Y	Y	N	U	Y	Y	Y	80%
Khademi et al. (2024) (38)	Y	Y	Y	Y	Y	U	Y	Y	Y	Y	90%
Leitão et al. (2024) (34)	Y	Y	Y	Y	Y	U	U	Y	Y	Y	80%
Taylor-Swanson et al. (2024) (27)	Y	Y	Y	Y	Y	N	N	Y	Y	Y	80%
Ratio	100%	100%	100%	100%	100%	13%	17%	100%	83%	100%	

C1, Is there congruity between the stated philosophical perspective and the research methodology? C2, Is there congruity between the research methodology and the research question or objectives? C3, Is there congruity between the research methodology and the methods used to collect the data? C4, Is there congruity between the research methodology and the representation and analysis of the data? C5, Is there congruity between the research methodology and the interpretation of the results? C6, Is there a statement locating the researcher culturally or theoretically? C7, Is there a statement of the influence of the researcher on the research, and vice versa, addressed? C8, Are participants and their voices adequately represented? C9, Is there ethical approval by an appropriate body? C10, Is there a relationship between the conclusions and analysis, or interpretation of the data? Y, yes; N, no; U, unclear; NA, not applicable.

### 3.3 Study characteristics

The key characteristics of the included studies are summarized in Table 3. All included studies were published between 2004 and 2024. The 23 included studies involved 808 participants from different countries including five from USA (14, 24–27), three from Taiwan, China (28–30), two each from China (31, 32), Portugal (33, 34), Sweden (35, 36), Iran (37, 38), one each from Mexico (39), Singapore (40), Canada (41), England (42), Australia (43), Korea (44), and Pakistan (45).

**Table 3:** Study characteristics.

Author(s) (year)	Country	Study design	Data collection method/methodology for data analysis	Participants	Aim(s)	Main findings
Chiang et al. (2009) (28)	Taiwan, China	Qualitative study	Semi-structured interviews	19 women (aged 49 to 68 years old)	To explore disturbance experiences and vaginal symptom self-management among menopausal women	Main themes: 1. Disturbance experiences of vaginal symptom; 2. Vaginal symptom self-management
Jeng et al. (2004) (29)	Taiwan, China	Grounded theory research	In-depth interviews/constant comparative method	12 menopausal women (mean age: 54 years)	To explore the experiences of regularly exercising	Main themes: 1. Perceiving continuous power; 2. Awareness of health crisis: Incurable chronic disease;3. Exercise selection; 4. Health becoming
Im et al. (2011) (24)	USA	Qualitative online forum study	Online forums/Im’s (56) feminist approach	13 Asian American women (aged 42 to 59 years old)	To explore the menopausal symptom experiences	Main themes: 1. Being conditioned; 2. becoming strong; 3. Appreciating; 4. without making a fuss; 5. quiet support.
Xiong et al. (2012) (31)	China	Qualitative study	Face-to-face semi-structured interviews/thematic analysis	52 menopausal patients (aged 38–62 years old)	To explore the self-management ability of menopausal patients and its influencing factors	Main themes: 1. some climacteric patients are irritable, often accompanied by complications; 2. lack of self-management knowledge and skills, and have misunderstandings; 3. Receiving professional guidance from medical staff was limited.
Doubova et al. (2012) (39)	Mexico	Exploratory qualitative analysis	Counseling group sessions/qualitative inductive thematic analysis	121 women (aged 45 to 59 years old)	To identify the changes in women’s discourse regarding their concerns and needs about the climacteric stage and self-care after they had participated in an integrative women-centered healthcare model with empowerment for self-care	Main themes: 1. Lack of information about changes during the climacteric stage and self-care; 2. Tradition: the climacteric stage as a taboo subject; 3. life’s changes and transitions: the complexity of the climacteric experience; 4. Stigma of menopause; 5. Relationship between the traditional gender role and the lack of self-care; 6. The climacteric as a natural stage; 7. Expectations for old age; 8. Empowerment and the change of awareness for self-care; 9. De-medicalization of the climacteric; 10. The richness of group work; 11. Empowerment as motivation to convey acquired knowledge.
Odiari et al. (2012) (14)	USA	Qualitative study	Open-ended questions during one-on-one interviews/content analysis	34 menopausal Ghanaian women (average age: 58.6 years)	To explore their sources of information, perceptions, attitudes, and self-management methods for alleviating menopausal symptoms	Main themes: 1. People who have experienced it; 2. It’s part of human growth; it’s a change; 3. I have to live with the experience and it really bothers me; 4. I did not do anything. I did not take any medicine; 5. When it happens, I just pour cold water on my body; 6. Once in a while I will take a pain killer; 7. The doctor prescribes medication for me and I apply Shea butter.
Lee et al. (2014) (30)	Taiwan, China	Grounded theory research	Face-to-face audio-taped interviews/constant comparative method	19 women (aged 45 to 67 years)	To generate a descriptive theoretical framework about the experiences of women who discontinued hormone replacement therapy	Main themes: 1. Relieving my discomforts safely; 2. Immediately discontinuing hormone replacement therapy--it would hurt my body; 3. Symptoms bothered me again; 4. negative emotions; 5. learning to let it go; 6. Trying to use non-hormone replacement therapy or products; 7. choosing safely therapies as first priority; 8. choose suitable non-hormone replacement therapy way; 9. Reuse hormone replacement therapy cautiously.
Mackey et al. (2014) (40)	Singapore	Descriptive, qualitative research	Semi-structured interview/thematic analysis	58 Chinese, Malay, and Indian Singaporean women (aged 40 to 60 years)	To explore knowledge, attitudes, and practices associated with the menopause transition	Main themes: 1. Knowledge; 2. Attitude; 3. Practice
McArthur et al. (2014) (41)	Canada	Interpretive description qualitative study	Individual interviews/Inductive content analysis	53 women (aged 40–62 years)	To identify enablers and barriers influencing adherence to regular exercise in middle-aged women who exercise.	Main themes: 1. Routine; 2. Intrinsic motivation; 3. Biophysical issues; 4. Psychosocial commitments; 5. Environmental factors; 6. Resources
Hardy et al. (2017) (42)	England	Qualitative investigation	Three open-ended questions/inductive thematic analysis	137 women (average age:54 years)	To explore women’s perspectives on what employers and managers should and should not do in relation to women going through the menopause at work.	Main themes: 1. employer/manager awareness; 2. employer/manager communication skills and behaviors; 3. Employer actions.
Bahri et al. (2017) (37)	Iran	Exploratory qualitative study	Semi-structured in-depth interviews/Conventional content analysis	21 women (aged 42–55 years old)	To explore the ways of managing sexual dysfunctions during the menopausal transition	Main themes: 1. Confronting the decline of libido; 2. Seeking strategies for coping; 3. Achieving problem-solving strategies
Herbert et al. (2020) (43)	Australia	Cross-sectional, qualitative research study	Semi-structured interviews/reflexive thematic analysis	32 participants (aged 46–69 years)	To determine women’s knowledge of menopause and its consequences, and their menopause-related healthcare experiences.	Main themes:1. Understanding of menopause and associated symptom; 2. Approaches to improve overall health at menopause; 3. Approaches to symptom management; 4. Menopause and healthcare; 5. Menopause-related resources
Kim et al. (2020) (44)	Korea	Qualitative study	FOCUS Group Interview/theory-guided thematic analysis	21 Korean postmenopausal women (aged 54 to 69 years)	To identify the factors that affect the maintenance of healthy lifestyle habits in postmenopausal women	Main themes: 1. Taking a step toward healthy behavior maintenance using intrinsic and extrinsic motivational factors; 2. Lifestyle rebalancing under the self-regulation employing internal and external reinforcement; 3. Failure to integrate the healthy habits into lifestyle; 4. Inappropriate supportive strategy.
Pimenta et al. (2020) (33)	Portugal	Mixed-methods study	Semi-structured in-depth interviews/content analysis	27 Portuguese women (aged 40–65 years)	To assess the suitability of the self-regulation model in explaining the menopausal experience.	Main themes: 1. Identify; 2. undefined identify; 3. Cause; 4. Control; 5. Undefined consequences; 6. Negative consequences; 7. Positive consequences
Asad et al. (2021) (45)	Pakistan	Qualitative exploratory design	In-depth interviews /content analysis	eleven women (aged 35–55 years)	To explore the perceptions and experiences of menopausal women	Main themes: 1. Bio-psycho-social challenges; 2. Misconceptions about menopause; Role of healthcare providers (HCP).
Ilankoon et al. (2021) (35)	Sweden	Qualitative exploratory research	Individual interviews /manifest and latent content analysis	20 postmenopausal women (aged 46–55 years)	To explore and describe menopausal experiences among women	Main themes: 1. Menopause is a natural stage of aging
Berin et al. (2022) (36)	Sweden	qualitative study	Individual semi-structured interviews/thematic analysis	15 postmenopausal women (aged 49 to 68 years)	To explore postmenopausal women’s experiences of participation in a resistance-training intervention to find barriers and motivators for the training.	Main themes: 1. Trigger-Hopes of symptom relief; 2. An evolving motivation as a driving force for change; 3. Finding new triggers
Kracht et al. (2022) (25)	USA	Qualitative study	Focus groups/Thematic analysis	27 Black women (mean age: 54 years)	To identify barriers and facilitators for participating in a lifestyle program	Main themes: 1. past experiences;2. menopause experiences; 3. Lifestyle program components; 4. Lifestyle program development& considerations
Cortes et al. (2024) (26)	USA	Qualitative descriptive study	5 focus groups/content analysis	29 midlife women (average age: 50.3 years)	To understand knowledge, attitudes, and experiences of the menopause transition	Main themes:1. menopause is a stage of life; 2. not wanting to become an old lady; 3. in our culture, we do not ask; 4. family dynamics; 5. each body is different; 6. Menopause self-management and treatment options; 7. information is power.
Zhang et al. (2024) (32)	China	Qualitative study	Semi-structured interviews/Colaizzi seven-step analysis method	17 women in the menopausal transition (aged 44 to 53 years)	To understand women′s experiences and health management needs during the menopausal transition	Main themes:1. physical and mental distress;2. coping strategies; 3. Perceived health risks and medical seeking experience; 4. Health management needs.
Khademi K et al. (2024) (38)	Iran	Exploratory qualitative study	9 focus group discussion/Graneheim and Lundman’s method	30 menopausal women (aged 45–65 years)	To explore the barriers to a healthy lifestyle among Iranian postmenopausal women.	Main themes: perceived lack of behavioral control as a barrier to a healthy lifestyle in post-menopause
Leitão et al. (2024) (34)	Portugal	Qualitative descriptive study	Semi-structured interviews/deductive-dominant content analysis	31 Portuguese postmenopausal women (aged 45–65 years)	To identify the successful cognitive and behavioral weight management strategies employed by postmenopausal women who effectively maintained a healthy weight during the menopausal transition	Main themes: 1. dietary choices; 2. Effortful inhibition; 3. Energy compensation; 4. engaging in physical activity/exercise; 5. food literacy; 6. information seeking; 7. intuitive eating; 8. planning content; 9. psychological self-care; 10. regulation: allowances; 11. regulation: restrictions; 12. regulation: rule setting; 13. Restraint; 14. Self-monitoring; 15. Stimulus control; 16. Support: help from others; 17. weight management aids
Taylor-Swanson et al. (2024) (27)	USA	Qualitative descriptive study	Focus group discussion/Conventional content analysis	Nine women (aged 41–55 years)	To gather women’s opinions about the menopause, provider access, and conventional and integrative health interventions for later use to develop a menopause Medical group visit	Main themes: 1. An interest in this topic; 2. Unfamiliar medical terms;3. relevant social factors; 4. Desired whole person care; 5. Interest in integrative health;6. barriers and facilitators to accessing healthcare.

Fifteen of the included studies used interviews (14, 28–37, 40, 41, 43, 45), four used focus groups (25–27, 38, 44), one used online forums (24), one used open-ended questions by online questionnaire (42), and one used counseling group sessions (39).

### 3.4 Synthetic results

We have identified six themes with fifteen sub-themes: Poor cognition (misconceptions about menopause and treatment, insufficient knowledge, lack of active health literacy, and lack of empowerment); physical restriction (medical condition and fatigue); restricted environment (limited resources and restrictions on amenities); impact of interpersonal circle (the influence of family support and understanding, social belonging, and lack of useful advice from experts); planning and adherence (setting solid plan and psycho-immune system); expectations for keeping health (perceived health benefits and health crisis concern), as shown in Table 4.

**Table 4:** Themes and sub-themes.

COM-B domain	Theme	Sub-theme	Sources
Psychological Capability	Poor recognition	Misconceptions about menopause and treatment	14, 24, 26, 28–30, 32, 34, 37, 39, 40, 43, 45
		Insufficient knowledge	25, 29, 31, 32, 39, 45
		Lack of active health literacy	14, 28, 29, 32, 40, 44
		Lack of empowerment	24, 25, 27, 34, 39
Physical capability	Physical restriction	Medical condition	25, 40, 41, 44
		Fatigue	41
Physical opportunity	Restricted environment	Limited resources	26, 36, 27, 38, 41, 44
		Restrictions on Amenities	32, 38, 41, 42, 44
Social opportunity	Impact of interpersonal circle	Influence of family support and understanding	24–26, 32, 34, 37, 38, 41, 44, 45
		Social belonging	25–27, 29, 32, 34–38, 41, 42, 44
		Lack of useful advice from experts	14, 27, 29, 31, 32, 44
Reflective motivation	Planning and adherence	Setting a solid plan	25, 34, 44
		Psycho-immune system	25, 29, 34, 35, 41, 42, 44
	Expectations for keeping healthy	Perceived health benefits	14, 26, 29, 35, 36, 40, 41, 44, 46
Automatic motivation	Expectations for keeping healthy	Health crisis concern	29, 44

#### 3.4.1 Poor cognition

##### 3.4.1.1 Misconceptions about menopause and treatment

Misconceptions regarding menopause were common and were mainly related to limited help-seeking behavior and reliance on informal sources of information (24). Some women perceived menopause as personal (40, 45) or a source of shame (26, 28), often viewing it as a stigma or a sign of aging (34, 39). As a result, they were reluctant to talk about it or to seek support from family members or healthcare providers. Talking about menopause issues (about sex) with their husband is “not good” or “embarrassed” (37). Otherwise, some women cited “law of nature” (32) to describe menopause as a reason for not taking any self-care strategies. Misconceptions about the treatment of menopausal symptoms, especially hormone replacement treatment, result in not taking medication. The majority of women in this study believed that taking hormones or synthetic hormones could cause “side effects” (26) like “breast cancer” (29, 30, 40, 43), or “to be addicted” (14).

*I haven’t discussed it around because it is very personal. (* *45* *) (p.204)*

*I generally don't open these issues (about sex) … I'm not even much open with my husband … Cuz in my family we think talking about these kinds of things is not good …, I'm embarrassed to talk about these things with my husband and so we don't talk about sex at all. (* *37* *) (p.182)*

##### 3.4.1.2 Insufficient knowledge

Data suggested that a lack of knowledge about menopause made them feel uncomfortable, stressed, and confused about how to take accurate self-management strategies for managing menopause (31). The majority of women do not know about “basic things” (45), do not know how to do or how to prevent or at least alleviate the symptoms they have (39), and do not understand all of the symptoms (25). They not only lack knowledge about the climacteric but they also frequently followed popular beliefs that were even a dubious guide and harmed them (39). Sufficient knowledge helped women to choose suitable self-management strategies (29). Women reported that there is a lot of knowledge on the Internet, but they do not know whether it’s true or false after reading it, and they usually choose a method that suits them and has little risk to try (32).

*No idea of what climacteric and the menopause are; what I know is very superficial. So, I know there are many things that happen to us; many times we don’t know what to do, or how to prevent or at least alleviate the symptoms we have … We do not know how to manage all these changes. (* *39* *) (p.566)*

##### 3.4.1.3 Lack of empowerment

Although they recognized their lack of knowledge, many women expressed a willingness to be empowered and a desire to learn about climacteric. They were aware that they were experiencing many changes they would have to live with but did not fully understand, often suffering due to this lack of information (25, 39). Women need more knowledge, more “empowerment” to say “yes, we can” (24, 25, 27, 39). Meanwhile, women stated that healthcare providers should set up a menopausal transition course for middle-aged women. It is better to obtain popular science education from the perspective of doctors so that they can prepare in advance to get through this period (34).

*I [have] so many symptoms, and I don’t understand all of it you know. I just would like to learn more about it. (* *25* *) (p.18)*

##### 3.4.1.4 Lack of active health literacy

Some women lacked a perceived threat to their health, which hindered them from exercising (44). They seek medical attention, such as consulting a doctor, only if there are severe symptoms or diseases (29, 32, 40). Furthermore, some women do not take any measures (14) or do not show willingness to seek medical treatment (28).

*Only if you know it is really bad, then I may consult a doctor. (* *40* *) (p.521)*

*I didn’t do anything. I didn’t take any medicine. After some time, the pains ceased on its own. (* *14* *) (p.567)*

#### 3.4.2 Physical restriction

##### 3.4.2.1 Medical condition

Menopausal women identified medical conditions, such as constitution of sensitivity (40), injury/ailment (41, 44), thyroid condition (44), arthritis, problem with lower back, surgeries (25), as barriers to follow the self-management strategies, such as physical activity, weight management, and exercise.

*My physical activity has been compromised because I have a back and ankle situation”. (* *41* *) (p.5)*

##### 3.4.2.2 Fatigue

‘Fatigue’ captured responses wherein women articulated simply being “too tired” to be active, which hindered regular exercise (41).

*… when I get home, I don’t feel like it anymore, cause I'm too tired. (* *41* *) (p.5)*

#### 3.4.3 Restricted environment

##### 3.4.3.1 Limited resources

Some women identified “lack of time” as a barrier to maintaining regular exercise or physical activity, often due to full-time employment, childcare responsibilities (27), numerous household chores (38, 44), conflicts with work schedules (36, 44). This lack of time also affects their ability to attend yearly physical check-ups and maintenance visits (27). In addition, financial constraints make some women reluctant to join gyms (41) or access services such as gynecology, acupuncture (27), and natural lubricants (26). Access to healthcare resources was also cited as a challenge, mainly due to issues related to location and availability (27).

*A lot of people I know don’t even have the time to get a yearly physical check, let alone maintenance visits like massage or chiropractic. (* *27* *) (p.9)*

##### 3.4.3.2 Restrictions on amenities

The built environment was described in terms of how the infrastructure surrounding the women’s work/home allowed for safe integration of exercise into transportation or leisure activity (41). Women felt that it was much harder to manage menopausal symptoms by taking a shower or washing hands to relieve hot flashes (38). The suffocating air inside the gym or terrible atmosphere may hinder women to exercise (44). In addition, when they travel outside, there is a lack of the condition to cook (38), and they end up breaking the rules (44). Warm/hot office environment is also an influencing factor for them to manage their symptoms (42). Another important part is medical-related facilities and construction; most of the respondents thought that the medical care and health welfare of middle-aged women should be strengthened, such as setting up menopausal transition clinics or menopausal transition mental accommodation rooms in the community and medical institutions (32).

*If there is such a clinic, I think women in their 40s and 40s can go to consult one Next, preparing in advance can also help us age more slowly. (N3) (* *32* *) (p.100)*

#### 3.4.4 Impact of interpersonal circle

##### 3.4.4.1 Influence of family support and understanding

Family members’ misunderstanding and misconceptions about menopause also affect women’s health self-management. Women were motivated to participate in a self-management program by their family’s encouragement, support, and supervision (25, 34, 41, 44). A major of women emphasize the need for husbands’ support and understanding. Furthermore, they think family members should be taught about the changes that women are experiencing (24, 26, 32, 45), especially about sexual relationships with their husbands, which hinder women from seeking strategies for resolution (37, 38).

*My husband doesn’t show me much support or love. I often dwell on this issue at night, which makes it hard for me to sleep and causes me to feel moody throughout the day. (Housewife, upscale area) (* *38* *) (p.7)*

##### 3.4.4.2 Social belonging

Peer support from colleagues who are also going through menopause (37), other menopausal women around them (29, 35, 37), close friends of the same age (32, 35), and accountability partners (25) were cited as key facilitators who positively influenced women’s motivation for health self-management and symptom management. This support helped by providing opportunities to share experiences (37), receive advice (37), and maintain regular exercise (29). In addition, socializing during exercise can also help menopausal women to maintain exercise for health self-management (36). In the process of social interaction, participants had talked about the intervention trial with their relatives and colleagues, which created pressure to keep up the regular resistance training or they felt accountable to the physiotherapist (36), peer pressure, coach reminder, personal trainer consulting, and being inspired by other people (44), who also motivated them while maintaining the program (34, 44). Another special factor is that the company of a pet is also a factor that promotes regular exercise (41). On the other hand, they emphasized the need for a support group to increase awareness and knowledge about menopause for self-management improvement (26) and need their managers’ understanding (42). Some women also expressed a need to talk with other women and share their experiences (27). However, increased social interactions unrelated to self-management sometimes hindered them from engaging in physical activity (38).

*Sometimes I get advice (from menopausal women around me) … they recommended me that an exercise program can be helpful … I listened to their advice and now I feel a little better. (* *37* *) (p.183)*

*When I have guests, I’m busy with catering and cleaning the house since morning, so I can’t do any physical activity. (* *38* *) (p.6)*

*I have to walk the dogs … I have to because I don't have a choice, which is a good thing. (* *41* *) (p.5)*

##### 3.4.4.3 Lack of useful advice from experts

Some participants reported that some providers provide unhelpful medical information, such as those not associated with their symptoms, and felt frustrated to listen to their doctor (27), and some doctors did not tell them to take self-management (14, 32, 44) or could not help them (29). Guidance from medical staff played little role, and only a few interviewees mentioned medical and nursing staff when asked about the way of knowledge acquired (31). Communication is needed in their community and between community people and their providers (27).

*The internal medicine doctor didn’t tell me to lose weight contrary to what I expected. I found that a bit strange, but it did make me slack off a bit. (* *44* *) (p.9)*

#### 3.4.5 Planning and adherence

##### 3.4.5.1 Setting solid plan

Setting a goal is one important part of planning, and setting a realistic goal based on previous experience with healthy lifestyle modifications and behavioral changes served as an enabling factor for the maintenance of a healthy lifestyle (25, 34, 44). After setting a goal, monitoring is another part to compare with the set goal, and helps to adjust the plan dynamically to achieve self-management (34, 44). But some respondents said that there will be disruptions in their daily routine to stay consistent because of family and daily routine (44).

*I think it would be easy if I had a goal. I once got rid of hypertension by exercising. I started participating in the program hoping to reduce my medication use if not completely stop it. With that goal in mind… or the goal to lose weight… I don’t want to be fat again. (* *44* *) (p.6)*

##### 3.4.5.2 Psycho-immune system

“Self-discipline” and “self-reflection” constituted the psycho-immune system. Reflection, such as diagnostic scans to identify cognitive viruses (bias/error), and self-discipline looked like antibody formation, clearing behavioral pathogens (procrastination/indulgence). Some women discovered the causes of their obesity by comparing, observing, and comparing with others, thereby promoting behavioral change (44). Participants reported that when holding a high level of self-discipline. Once they beat the temptation and overcome it, they felt achieved (44) and were more motivated to help lifestyle modification and weight self-management strategies (29, 34, 35, 42, 44). However, some women had lack of self-discipline to hinder weight management because of the accessibility of things of interest (25, 44). “Self-sabotage” was evident when women described putting obstacles in the way to avoid exercise (41).

*It’s more important to be self-motivated. If you don’t want to exercise, no matter how hard people encourage or push you, it just won’t work. (* *29* *) (p.451)*

#### 3.4.6 Expectations for keeping healthy

##### 3.4.6.1 Perceived health benefits

Menopausal women stated they identified perceived health benefits as an enabling factor within the theme of physical wellbeing, which enables adherence to the self-management strategies. They found that some self-management strategies make them “felt better” (14, 26, 29, 36), “no stiff, no pain or illness,” “more beautiful and confident” (29), “good shape” (36, 44), “stronger” (36), also “relieve stress” (41) or, of course that can happen but it is a way to keep their health and all (36). Not only did it let them feel better, but it also resolved the symptom problem, such as “sleep problem” (35, 40) “perceived health benefits,” perceived health benefits pertains to the motivation attached to resistance training (36), very low energy diet (46).

*When you discover the importance of exercise, you will do it yourself without being pushed by anybody. (* *29* *) (p.451)*

##### 3.4.6.2 Health crisis concern

Triggered by physiological discomfort, incurable chronic disease, aging, and deterioration, menopausal women begin to have health concerns, and they are motivated to maintain a healthy lifestyle (29, 44).

*I have diabetes… I love sweets, but I didn’t know I had diabetes. My father had diabetes, but it didn’t run in my families… Anyways, I really needed to lose weight. (* *44* *) (p.6)*

### 3.5 Reflection

**Figure 2:**
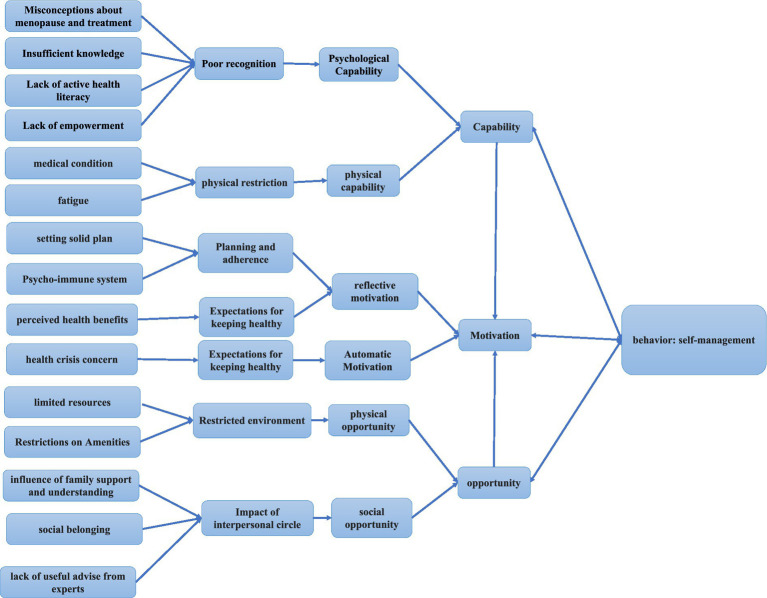
Map to COM-B model.

We synthesized results (six themes and fifteen sub-themes) according to the COM-B model. We mapped the themes of poor cognition (misconceptions about menopause and treatment, insufficient knowledge, lack of active health literacy, and empowerment) and physical restriction (medical condition and fatigue). We mapped the restricted environment (limited resources and restrictions on amenities) and the impact of interpersonal circle (influence of family support and understanding, social belonging, and lack of useful advice from experts) to the opportunities section. We mapped planning and adherence (setting a solid plan and psycho-immune system) and the expectations for keeping health (perceived health benefits and health crisis concern) to the motivation section (Table 4; Figure 2). The findings were mapped according to the COM-B model, and then we combined the pooled results with the corresponding nine interventions to propose clinical interventions (20).

## 4 Discussion

To the knowledge of the authors of this study, this review is the first to explore the experiences, facilitators, and challenges of self-management behaviors in perimenopausal women, although growing focus on self-management in menopausal transitional and postmenopausal women among researchers. Thus, this research extends the content and field of menopausal transitional and postmenopausal women’s self-management.

Similar to previous results from quantitative studies (19, 47), our study found that the menopausal transitional and postmenopausal women had a prominent lack of accurate and comprehensive knowledge about menopause and treatment; similar results were found for factors related to self-perceived health and time (48). Therefore, considering the quantitative studies and qualitative studies’ results, health professionals may focus on how to meet the needs of knowledge about menopause and its treatment in future studies. Poor cognition, expectations for keeping healthy, and a restricted environment are the challenges faced by menopausal transitional and postmenopausal women. The expectation of staying healthy is the driving force for self-management behaviors. Impact of interpersonal circle, planning, and adherence are the important influencing factors of maintaining self-management behavior in perimenopausal women. The results of a study by Wang et al. (49) were similar to our study and showed that self-management of breast cancer-related lymphedema was associated with personal knowledge and experience, personal health beliefs, self-regulation skills and abilities, social influences, and support. Previous studies have shown that health literacy, social support, and self-management efficacy also affect self-efficacy among older adult patients with chronic diseases (50). However, our study found that physical restriction and a restricted environment also hindered the self-management behaviors in menopausal transitional and postmenopausal women, for the reasons of special symptoms such as hot flashes management needs and health-related behaviors, such as the environment required for physical activity. Another difference was that menopausal women express a great need for support and understanding from their husbands because of the sexual problems challenge and living or dwelling together for long periods of time. In the traditional culture of society, women often assume the important task of caring for family members. Therefore, considering how to meet women’s needs for support and understanding from their husbands and the environmental needs, including medical-related facilities, for implementing self-management related behaviors may be the focus and direction of further research.

We combined the results with the nine corresponding interventions to map the results to the COM-B model (20), and four interventions, which included education, training, persuasion, and environmental restructuring to provide clinical consultation and advice, were selected. Education promotion strategies are not just for perimenopausal women, but also for family members and related healthcare providers, which not only meet the women’s need for knowledge about menopause, and the needs for understanding and support from family members, but also improve the situation regarding the unhelpful information from healthcare providers. But the limitation of healthcare resources makes it difficult for women and their family members to meet their information and knowledge needs. Previous studies have taken measures such as online digital storytelling tools to empower women to seek health professional advice (51). Vollrath et al. reviewed self-management eHealth solutions for menopause and found that most eHealth-based information tools needed to be improved in terms of participatory design, readability, and content balance (52), especially to evaluate accessibility, acceptability, understanding, and usability in women of all education levels and socioeconomic conditions (53). At present, there is a lack of relevant education research on medical staff and family members. Training interventions may focus on women who have other medical conditions, which may hinder self-management behavior, such as injury, thyroid condition, arthritis, problems with the lower back, and surgeries aimed at imparting skills or attention for self-management behaviors. Environmental restructuring must be conducted through the improvement of the surrounding environment and making it convenient and comfortable. In this process, environmental limitations and inadequate support can negatively affect self-regulation, thereby hindering the development of healthy lifestyle habits.

Environmental restructuring included family and social support systems and the medical environment, such as community resources. Previous studies showed that family support was most associated with the severity of menopause-related symptoms (54, 55), but there are a few studies on the effect of family support on the self-management behavior of perimenopausal women. Studies have highlighted the importance of empowerment to empower perimenopausal women to become experts in their own self-management (10). Furthermore, community health service centers should carry out public awareness activities on menopause, improve women’s health literacy, develop a healthy lifestyle and self-management for all, and establish groups to enable mutual support, empowerment, and encouragement among perimenopausal or postmenopausal women. Finally, through persuasive measures, such as the communication skills of medical workers and health behavior management specialists, they can guide menopausal women to understand the seriousness of self-management and increase their motivation for self-management.

This study included menopausal transitional and postmenopausal women from several countries to understand their common problem and the influencing factors with self-management, and analyzed the findings using the COM-B model. However, this study only included Chinese and English studies, and studies in other languages were not included. Some studies were excluded because they aimed at self-management behaviors, including menopausal transitional and postmenopausal women accompanied by other diseases, such as autism, osteoporosis, etc. Therefore, we may not be able to provide a comprehensive experience of self-management and influencing factors in perimenopausal or postmenopausal women. Finally, as this review only included relevant published studies, there may also be publication bias that affects the generalization of the results of this study.

Clinical recommendations are concluded according to the results. First, clinical healthcare providers should recognize the need for knowledge among menopausal transitional and postmenopausal women regarding menopause, the treatment options, and self-management behaviors. Providers should offer detailed, individualized management plans for menopausal transitional and postmenopausal women. Second, we should focus on the important role of community health service centers in menopausal transitional and postmenopausal women’s self-management, establish menopausal transitional and postmenopausal outpatient clinics, and improve the diagnosis, treatment, and empowerment skills of menopausal management of healthcare workers in community health service centers. Finally, we should emphasize the importance of family support and social relationships in motivating self-management behaviors in menopausal women. We may consider incorporating this into future intervention studies to promote self-management behaviors.

## 5 Conclusion

This study synthesized the experiences, motivations, and challenges of self-management among menopausal transitional and postmenopausal women. The results suggest that knowledge and empowerment, family, and social support are particularly important motivators for self-management during perimenopausal and postmenopausal stages. Based on the COM-B model analysis, we conclude that meeting perimenopausal and postmenopausal women’s knowledge needs, improving the knowledge and skills of healthcare providers, and reinforcing supportive environments for self-management, such as promoting physical activity, improving access to community health services, and fostering interpersonal support networks, are effective interventions to promote women’s self-management.

## Data Availability

The original contributions presented in the study are included in the article/Supplementary material, further inquiries can be directed to the corresponding authors.

## References

[1] WHO Scientific Group on Research on the Menopause in the 1990s & World Health Organization. Research on the menopause in the 1990s. Report of a WHO scientific group. World Health Organ Tech Rep Ser. (1996) 866:1–107. 8942292

[2] Jia Y Zhou Z Cao X Hu W Xiang F Xiong LW. Incidence of perimenopausal syndrome in Chinese women aged 40 to 65 years:a Meta-analysis. Chin Gen Pract. (2023) 26:4080–8. doi: 10.12114/J.Issn.1007-9572.2023.0303

[3] Nachtigall LE. The symptoms of perimenopause. Clin Obstet Gynecol. (1998) 41:921–7. doi: 10.1097/00003081-199812000-00018, PMID: 9917947

[4] Marjoribanks J Farquhar C Roberts H Lethaby A Lee J. Long-term hormone therapy for perimenopausal And postmenopausal women. Cochrane Database Syst Rev. (2017) 1:Cd004143. doi: 10.1002/14651858.CD004143.Pub5 28093732 PMC6465148

[5] Li Q Gu J Huang J Zhao P Luo C. "they see me as mentally ill": the stigmatization experiences of Chinese menopausal women in the family. BMC Womens Health. (2023) 23:185. doi: 10.1186/S12905-023-02350-Y, PMID: 37076835 PMC10116657

[6] O'Neill MT Jones V Reid A. Impact of menopausal symptoms on work and careers: a cross-sectional study. Occup Med (Lond). (2023) 73:332–8. doi: 10.1093/Occmed/Kqad078 37542726 PMC10540666

[7] Geukes M Van Aalst MP Robroek SJ Laven JS Oosterhof H. The impact of menopause on work ability in women with severe menopausal symptoms. Maturitas. (2016) 90:3–8. doi: 10.1016/J.Maturitas.2016.05.001, PMID: 27282787

[8] D'Angelo S Bevilacqua G Hammond J Zaballa E Dennison EM Walker-Bone K. Impact of menopausal symptoms on work: findings from women in the health and employment after fifty (HEAF) study. Int J Environ Res Public Health. (2022) 20:295. doi: 10.3390/Ijerph20010295 36612616 PMC9819903

[9] Jaspers L Daan NM Van Dijk GM Gazibara T Muka T Wen KX . Health in middle-aged and elderly women: a conceptual framework for healthy menopause. Maturitas. (2015) 81:93–8. doi: 10.1016/J.Maturitas.2015.02.010, PMID: 25813865

[10] Hickey M Lacroix AZ Doust J Mishra GD Sivakami M Garlick D . An empowerment model for managing menopause. Lancet. (2024) 403:947–57. doi: 10.1016/S0140-6736(23)02799-X, PMID: 38458214

[11] Donison V Chesney TR Wills A Santos B Mclean B Alqurini N . Self-management interventions for issues identified in a geriatric assessment: a systematic review. J Am Geriatr Soc. (2022) 70:1268–79. doi: 10.1111/Jgs.17601, PMID: 34902156

[12] Grady PA Gough LL. Self-management: a comprehensive approach to management of chronic conditions. Am J Public Health. (2014) 104:E25–31. doi: 10.2105/Ajph.2014.302041 24922170 PMC4103232

[13] Lorig KR Holman H. Self-management education: history, definition, outcomes, and mechanisms. Ann Behav Med. (2003) 26:1–7. doi: 10.1207/S15324796abm2601_01, PMID: 12867348

[14] Odiari EA Chambers AN. Perceptions, attitudes, and self-management of natural menopausal symptoms in Ghanaian women. Health Care Women Int. (2012) 33:560–74. doi: 10.1080/07399332.2012.655393, PMID: 22577742

[15] Lobo RA Davis SR De Villiers TJ Gompel A Henderson VW Hodis HN . Prevention of diseases after menopause. Climacteric. (2014) 17:540–56. doi: 10.3109/13697137.2014.933411, PMID: 24969415

[16] Hulteen RM Marlatt KL Allerton TD Lovre D. Detrimental changes in health during menopause: the role of physical activity. Int J Sports Med. (2023) 44:389–96. doi: 10.1055/A-2003-9406, PMID: 36807278 PMC10467628

[17] Stanzel KA Hammarberg K Fisher J. Experiences of menopause, self-management strategies for menopausal symptoms and perceptions of health care among immigrant women: a systematic review. Climacteric. (2018) 21:101–10. doi: 10.1080/13697137.2017.1421922, PMID: 29345497

[18] Chiu HH Tsao LI Liu CY Lu YY Shih WM Wang PH. The perimenopausal fatigue self-management scale is suitable for evaluating perimenopausal Taiwanese women's vulnerability to fatigue syndrome. Healthcare (Basel). (2021) 9:336. doi: 10.3390/Healthcare9030336, PMID: 33809807 PMC8002518

[19] Xian D. Research on constructing self-management program for perimenopausal women based on evidence. Zunyi: Zunyi Medical University (2023).

[20] Michie S Van Stralen MM West R. The behaviour change wheel: a new method for characterising and designing behaviour change interventions. Implement Sci. (2011) 6:42. doi: 10.1186/1748-5908-6-42, PMID: 21513547 PMC3096582

[21] Lockwood C Munn Z Porritt K. Qualitative research synthesis: methodological guidance for systematic reviewers utilizing meta-aggregation. Int J Evid Based Healthc. (2015) 13:179–87. doi: 10.1097/Xeb.0000000000000062, PMID: 26262565

[22] Tong A Flemming K Mcinnes E Oliver S Craig J. Enhancing transparency in reporting the synthesis of qualitative research: ENTREQ. BMC Med Res Methodol. (2012) 12:181. doi: 10.1186/1471-2288-12-181 23185978 PMC3552766

[23] Thomas J Harden A. Methods for the thematic synthesis of qualitative research in systematic reviews. BMC Med Res Methodol. (2008) 8:45. doi: 10.1186/1471-2288-8-45, PMID: 18616818 PMC2478656

[24] Im EO Lee SH Chee W. "being conditioned, yet becoming strong": Asian American women in menopausal transition. J Transcult Nurs. (2011) 22:290–9. doi: 10.1177/1043659611404429, PMID: 21519062

[25] Kracht CL Romain JS Hardee JC Santoro N Redman LM Marlatt KL. “It just seems like people are talking about menopause, but nobody has a solution”: a qualitative exploration of menopause experiences and preferences for weight management among black women. Maturitas. (2022) 157:16–26. doi: 10.1016/J.Maturitas.2021.11.005, PMID: 35120668 PMC8969889

[26] Cortes YI Cazales A Duran M Trocel L. Información es Poder (information is power): menopause knowledge, attitudes, and experiences in midlife Latinas. Menopause. (2023) 30:1271–2. doi: 10.1097/GME.0000000000002288 PMC11613912 39623354

[27] Taylor-Swanson L Stoddard K Fritz J erson B Cortez M Conboy L . Midlife women's menopausal transition symptom experience and access to medical and integrative health care: informing the development of MENOGAP. Glob Adv Integ Med Health. (2024) 13:1–13. doi: 10.1177/27536130241268355, PMID: 39092447 PMC11292722

[28] Chiang H Jou H Kao C Tsao L. Disturbance experiences and vaginal symptom self-management among menopausal women. J Nurs. (2009) 56:43–51. 19222000

[29] Jeng C Yang S-H Chang P-C Tsao L-I. Menopausal women: perceiving continuous power through the experience of regular exercise. J Clin Nurs. (2004) 13:447–54. doi: 10.1046/J.1365-2702.2003.00878.X 15086631

[30] Lee PS Lee CL Hu ST Tsao LI. Relieving my discomforts safely: the experiences of discontinuing HRT among menopausal women. J Clin Nurs. (2014) 23:2481–9. doi: 10.1111/Jocn.12429, PMID: 24351027

[31] Xiong T. Qualitative research on self-management ability of menopausal patients. Chin J Coal Indust Med. (2012) 15:389–90.

[32] Zhang Y Geng L Liu Q Deng X Wan Y Wan C. Women’s experiences and health management neds during the menopausal transition:a qualitative study. J Nurs Sci. (2024) 39:98–101. doi: 10.3870/J.Issn.1001-4152.2024.18.098

[33] Pimenta F Ramos MM Silva CC Costa PA Maroco J Leal I. Self-regulation model applied to menopause: a mixed-methods study. Climacteric. (2020) 23:84–92. doi: 10.1080/13697137.2019.1640196, PMID: 31365272

[34] Leitão M Pérez-López FR Marôco J Pimenta F. Cognitive and Behavioral weight management strategies during the menopausal transition: insights from the menopause and weight loss (ME-WEL) project. Maturitas. (2024) 187:108060. doi: 10.1016/J.Maturitas.2024.108060 38959752

[35] Ilankoon IMPS Samarasinghe K Elgán C. Menopause is a natural stage of aging: a qualitative study. BMC Womens Health. (2021) 21:1–9. doi: 10.1186/S12905-020-01164-6 33522914 PMC7849153

[36] Berin E Spetz Holm A-C Hammar M Lindh-Åstr L Berterö C. Postmenopausal women's experiences of a resistance training intervention against vasomotor symptoms: a qualitative study. BMC Womens Health. (2022) 22:1–13. doi: 10.1186/S12905-022-01900-0 35907840 PMC9338607

[37] Bahri N Latifnejad Roudsari R Azimi Hashemi M. “Adopting self-sacrifice”: how Iranian women cope with the sexual problems during the menopausal transition? An exploratory qualitative study. J Psychosom Obstet Gynecol. (2017) 38:180–8. doi: 10.1080/0167482X.2016.1216962 27626135

[38] Khademi K Kaveh MH Nazari M Asadollahi A. Perceived lack of behavioral control is a barrier to a healthy lifestyle in post-menopause: a qualitative study. J Health Popul Nutr. (2024) 43:180. doi: 10.1186/S41043-024-00674-5, PMID: 39501414 PMC11539316

[39] Doubova SV Infante-Castaneda C Martinez-Vega I Perez-Cuevas R. Toward healthy aging through empowering self-care during the climacteric stage. Climacteric. (2012) 15:563–72. doi: 10.3109/13697137.2011.635824 22206414

[40] Mackey S Teo SSH Dramusic V Lee HK Boughton M. Knowledge, attitudes, and practices associated with menopause: a multi-ethnic, qualitative study in Singapore. Health Care Women Int. (2014) 35:512–28. doi: 10.1080/07399332.2013.801482, PMID: 23862640

[41] Mcarthur D Dumas A Woodend K Beach S Stacey D. Factors influencing adherence to regular exercise in middle-aged women: a qualitative study to inform clinical practice. BMC Womens Health. (2014) 14:49. doi: 10.1186/1472-6874-14-49 24666887 PMC3975263

[42] Hardy C Griffiths A Hunter MS. What do working menopausal women want? A qualitative investigation into women's perspectives on employer and line manager support. Maturitas. (2017) 101:37–41. doi: 10.1016/J.Maturitas.2017.04.011, PMID: 28539167

[43] Herbert D Bell RJ Young K Brown H Coles JY Davis SR. Australian women’s understanding of menopause and its consequences: a qualitative study. Climacteric. (2020) 23:622–8. doi: 10.1080/13697137.2020.1791072, PMID: 32705886

[44] Kim HR Yang HM. Facilitators and inhibitors of lifestyle modification and maintenance of Korean postmenopausal women: revealing conversations from focus group interview. Int J Environ Res Public Health. (2020) 17:1–20. doi: 10.3390/Ijerph17218178 PMC7663947 33167466

[45] Asad N Somani R Peerwani N Pirani S Zuberi N Andrades M . “I am not the person I used to be”: perceptions and experiences of menopausal women living in Karachi, Pakistan. Post Reprod Health. (2021) 27:199–207. doi: 10.1177/20533691211060099 34806468

[46] Harper C Maher J Hsu M Grunseit A Seimon R Sainsbury A. Postmenopausal women's experiences of weight maintenance following a very low energy diet. Obes Sci Pract. (2023) 9:305–19. doi: 10.1002/Osp4.654, PMID: 37287516 PMC10242258

[47] Bastos-Silva Y Aguiar LB Pacello P Baccaro LF Orcesi Pedro A Costa-Paiva L. Self-care agency and associated factors in postmenopausal women. Menopause. (2021) 28:1369–73. doi: 10.1097/Gme.0000000000001852, PMID: 34469935

[48] Cittadini N Basilici Zannetti E Iovino P De Maria M D'Angelo D Pennini A . Factors influencing self-care in postmenopausal women with osteoporosis: the Guardian angel® multicentric longitudinal study. Maturitas. (2022) 161:7–11. doi: 10.1016/J.Maturitas.2022.01.013, PMID: 35688499

[49] Wang Y Wei T Li M Wu P Qiang W Wang X . Factors influencing the self-management of breast Cancer-related lymphedema: a Meta-synthesis of qualitative studies. Cancer Nurs. (2024). doi: 10.1097/Ncc.0000000000001340 38704740

[50] Shao YJ Duan XC Xu XJ Guo HY Zhang ZY Zhao S . Latent profile and determinants of self-management Behaviors among older adult patients with chronic diseases: a cross-sectional study. Front Public Health. (2025) 13:1506545. doi: 10.3389/Fpubh.2025.1506545 39975786 PMC11835868

[51] Cumming GP Currie HD Moncur R Lee AJ. Web-based survey on the effect of digital storytelling on empowering women to seek help for urogenital atrophy. Menopause Int. (2010) 16:51–5. doi: 10.1258/Mi.2010.010004, PMID: 20729493

[52] Vollrath S Theis S Kolokythas A Janka H Schleich S Moreth J . Self-management ehealth solutions for menopause—a systematic scoping review. Climacteric. (2024) 27:255–68. doi: 10.1080/13697137.2024.2334035, PMID: 38685754

[53] Hammarberg K Bandyopadhyay M Nguyen H Cicuttini F Stanzel KA Brown H . Development and evaluation of 4 short, animated videos for women in midlife promoting positive health behaviors: survey study. Interact J Med Res. (2024) 13:E60949. doi: 10.2196/60949, PMID: 39621404 PMC11650076

[54] Zhao D Liu C Feng X Hou F Xu X Li P. Menopausal symptoms in different substages of perimenopause and their relationships with social support and resilience. Menopause. (2019) 26:233–9. doi: 10.1097/Gme.0000000000001208, PMID: 30252803

[55] Wang J Lin Y Gao L Li X He C Ran M . Menopause-related symptoms and influencing factors in Mosuo, Yi, and Han middle-aged women in China. Front Psychol. (2022) 13:763596. doi: 10.3389/Fpsyg.2022.763596, PMID: 35756261 PMC9226393

[56] Im EO. A feminist approach to research on menopausal symptom experience. Fam Commun Health. (2007) 30:S15–23. doi: 10.1097/00003727-200701001-00004 17159627

