# Supplementary material for: Menopausal transitional and postmenopausal women’s voices: “what influences their adherence to self-management”: a systematic review and meta-synthesis of qualitative studies
Source: Front Public Health. 2025 Oct 29;13:1653806. doi: 10.3389/fpubh.2025.1653806 (PMC12605042; doi:10.3389/fpubh.2025.1653806)
Supplement: Supplementary file 1 [file Supplementary_file_1.docx]

| **Examples of search strategy** | | |
| --- | --- | --- |
| **PubMed** | | |
| #1 | (("Perimenopause"[Mesh]) OR ( "Menopause"[Mesh] OR "Postmenopause"[Mesh] OR "Premenopause"[Mesh] )) OR "Climacteric"[Mesh] | 68432 |
| #2 | ((((perimenopause[Title/Abstract]) OR (menopause[Title/Abstract])) OR (climacteric[Title/Abstract])) OR (Postmenopause[Title/Abstract])) OR (Premenopause[Title/Abstract]) | 43449 |
| #3 | #1 OR #2 | 86553 |
| #4 | (("Self-Management"[Mesh]) OR "Self Care"[Mesh]) OR ( "Self Efficacy"[Mesh] OR "Self-Examination"[Mesh] OR "Self Medication"[Mesh] OR "Self-Assessment"[Mesh] OR "Self Administration"[Mesh] OR "Self-Testing"[Mesh] OR "Self-Directed Learning as Topic"[Mesh] OR "Self-Control"[Mesh] OR "Emotional Regulation"[Mesh] ) | 133802 |
| #5 | ((((((((((((((((self-management[Title/Abstract]) OR (self?care[Title/Abstract])) OR (self?help[Title/Abstract])) OR (self?manag*[Title/Abstract])) OR (self?admin*[Title/Abstract])) OR (self?monitor*[Title/Abstract])) OR (self?medicat*[Title/Abstract])) OR (self-nurs*[Title/Abstract])) OR (self -control[Title/Abstract])) OR (Disease Management[Title/Abstract])) OR (lifestyle[Title/Abstract])) OR (syndrome manage*[Title/Abstract])) OR (symptom manage*[Title/Abstract])) OR (seeking help[Title/Abstract])) OR (adherence[Title/Abstract])) OR (self-determinant*[Title/Abstract])) OR (lifestyle[Title/Abstract]) | 497008 |
| #6 | #4 OR #5 | 585323 |
| #7 | ((((("Interviews as Topic"[Mesh]) OR "Focus Groups"[Mesh]) OR "Narration"[Mesh]) OR "Qualitative Research"[Mesh]) OR "Personal Narratives as Topic"[Mesh]) OR "Grounded Theory"[Mesh] | 188661 |
| #8 | (((((((((("semi-structured"[Title/Abstract]) OR (semistructured[Title/Abstract])) OR (unstructured[Title/Abstract])) OR ("in-depth"[Title/Abstract])) OR (indepth[Title/Abstract])) OR ("face-to-face"[Title/Abstract])) OR (interview*[Title/Abstract])) OR (discussion*[Title/Abstract])) OR ("focus group"[Title/Abstract])) OR ("focus groups"[Title/Abstract])) OR (qualitative[Title/Abstract]) | 1328780 |
| #9 | #7 OR #8 | 1359022 |
| #10 | #3 AND #6 AND #9 | 554 |
| **Embase** | | |
| #1 | (perimenopause:ab,ti OR 'perimenopause'/exp OR 'menopause'/exp OR 'climacteric'/exp OR 'postmenopause'/exp OR postmenopause:ab,ti OR menopaus:ab,ti OR premenopause:ab,ti) OR 'premenopause'/exp | 154150 |
| #2 | 'self management':ab,ti OR 'self care':ab,ti OR 'self care'/exp OR 'self management'/exp OR 'self medication'/exp OR 'self control scale'/exp OR 'self medication':ab,ti OR 'self control':ab,ti OR 'self administration':ab,ti OR 'self administration'/exp OR self?monitor*:ab,ti OR self?help:ab,ti OR self?admin*:ab,ti OR 'self nurs*':ab,ti OR 'disease managemen':ab,ti OR 'self -control':ab,ti OR 'seeking help':ab,ti OR adherence:ab,ti OR 'self determinant*':ab,ti OR lifestyle:ab,ti | 681958 |
| #3 | ‘focus groups’/exp OR ‘focus groups’:ab,ti OR ‘qualitative research’:ab,ti OR ‘qualitative research’/exp OR ‘grounded theory’:ab,ti OR ‘grounded theory’/exp OR ‘semi structured interview’:ab,ti OR ‘in depth interview’:ab,ti OR ‘face-to-face interview’:ab,ti OR ‘focus group’:ab,ti | 3441824 |
| #4 | #1 AND #2 AND #3 | 717 |
| **Medline(via Ovid)** | | |
| #1 | Premenopause.mp. or Menopause/ or Premenopause/ | 39564 |
| #2 | Perimenopause.mp. or Menopause/ or Perimenopause/ | 33539 |
| #3 | Postmenopause.mp. or Menopause/ or Postmenopause/ | 59721 |
| #4 | Climacteric.mp. or Climacteric/ | 8126 |
| #5 | Menopause/ or Menopause.mp. | 54024 |
| #6 | #1 OR #2 OR #3 OR #4 OR #5 | 86418 |
| #7 | self-management.mp. or Self Care/ or Self-Management/ | 61343 |
| #8 | self?help.mp. | 18 |
| #9 | self?manag*.mp. | 69 |
| #10 | self?admin*.mp. | 110 |
| #11 | self?monitor*.mp. | 31 |
| #12 | self?medicat*.mp. | 31 |
| #13 | self-nursing.mp. | 29 |
| #14 | Self-Control/ or self -control.mp. | 10861 |
| #15 | self-determinant*.mp. | 161 |
| #16 | symptom manage*.mp. | 9026 |
| #17 | seeking help.mp. or Help-Seeking Behavior/ | 4505 |
| #18 | Medication Adherence/ or adherence.mp. or Guideline Adherence/ or "Treatment Adherence and Compliance"/ | 221706 |
| #19 | #7 OR #8 OR #9 #10 or #11 or #12 or #13 or #14 or #15 or #16 or #17 OR #18 | 299584 |
| #20 | Qualitative Research/ or qualitative.mp. | 405574 |
| #21 | Focus Groups.mp. or Focus Groups/ | 62393 |
| #22 | Grounded Theory.mp. or Grounded Theory/ | 17243 |
| #23 | Interview/ or Qualitative Research/ or Interviews.mp. | 384532 |
| #24 | Interviews as Topic/ or semi-structured.mp. | 147819 |
| #25 | Narration.mp. or Narration/ | 12029 |
| #26 | #20 OR #21 OR #22 OR #23 OR #24 OR #25 | 649199 |
| #27 | #6 and #19 and #26 | 95 |
| **Web of science core collection** | | |
| #1 | TS=(Perimenopause OR Menopause OR Postmenopause OR Premenopause OR Climacteric) | 49788 |
| #2 | TS=(self-management OR self?care OR self?help OR self?manag* OR self?admin* OR self?monitor* OR self?medicat* OR self-nursing OR self-control OR self-determinant* OR (symptom manage*) OR (seeking help) OR Adherence) | 610541 |
| #3 | TS=(qualitative study OR qualitative research OR qualitative method OR interview* OR Focus Groups OR Grounded Theory OR Narration) | 1745944 |
| #4 | #1 AND #2 AND #3 | 242 |
| #2 | TS=(self-management OR self?care OR self?help OR self?manag* OR self?admin* OR self?monitor* OR self?medicat* OR self-nursing OR self-control) | 88695 |
| #3 | TS=(qualitative study OR qualitative research OR qualitative method OR interview* OR experience* OR feeling* OR perception* OR attitude*) | 7220096 |
| #4 | #1 AND #2 AND #3 | 391 |
| **CINAHL(via EBSCO)** | | |
| #1 | XB (perimenopause or perimenopausal or premenopause or pre-menopause or pre-menopausal) OR XB (menopause or menopausal or perimenopause or perimenopausal) OR XB (postmenopause or post-menopausal or post-menopause) OR XB (premenopause or 'peri menopause') OR XB (climacteric or menopause or menopausal or perimenopause or perimenopausal) | 20,232 |
| #2 | XB (adherence or compliance or nonadherence or noncompliance or treatment adherence or treatment compliance) OR XB (self-management or self-care or self-regulation or self-monitoring) OR XB self help OR XB (self-monitoring or self monitoring or self tracking) OR XB (self-administration or self-supervised or self-administering or self-managed) OR XB (symptom management or symptom control or symptom relief) OR XB (seeking help or help-seeking) | 192,841 |
| #3 | XB (qualitative study or qualitative research or qualitative methods or interview) OR XB qualitative methods OR XB interview* OR XB (semi-structured interviews or semi structured interviews) OR XB Focus Groups OR XB Grounded Theory | 349,179 |
| #4 | #1 AND #2 AND #3 | 115 |
|  | *PsycInfo(via EBSCO)* |  |
| #1 | XB (perimenopause or perimenopausal or premenopause or pre-menopause or pre-menopausal) OR XB (menopause or menopausal or perimenopause or perimenopausal) OR XB (postmenopause or post-menopausal or post-menopause) OR XB (premenopause or 'peri menopause') OR XB (climacteric or menopause or menopausal or perimenopause or perimenopausal) | 5934 |
| #2 | XB (adherence or compliance or nonadherence or noncompliance or treatment adherence or treatment compliance) OR XB (self-management or self-care or self-regulation or self-monitoring) OR XB self help OR XB (self-monitoring or self monitoring or self tracking) OR XB (self-administration or self-supervised or self-administering or self-managed) OR XB (symptom management or symptom control or symptom relief) OR XB (seeking help or help-seeking) | 166,832 |
| #3 | XB (qualitative study or qualitative research or qualitative methods or interview) OR XB qualitative methods OR XB interview* OR XB (semi-structured interviews or semi structured interviews) OR XB Focus Groups OR XB Grounded Theory | 513,885 |
| #4 | #1 AND #2 AND #3 | 56 |
|  | **SCOPUS** |  |
| #1 | TITLE-ABS-KEY ( perimenopause OR perimenopausal OR premenopause OR pre-menopause OR pre-menopausal OR menopause OR menopausal OR postmenopause OR post-menopausal OR post-menopause OR 'peri AND menopause' OR climacteric ) | 84396 |
| #2 | TITLE-ABS-KEY ( adherence OR compliance OR nonadherence OR noncompliance OR self-management OR self-care OR self-regulation OR self-monitoring OR self AND help OR self-monitoring OR self AND monitoring OR self AND tracking OR self-administration OR self-supervised OR self-administering OR self-managed OR ( symptom AND management ) OR ( symptom AND control ) OR ( symptom AND relief ) OR ( seeking AND help ) OR help-seeking ) | 149513 |
| #3 | TITLE-ABS-KEY((qualitative study) or (qualitative research) or (qualitative methods) or interview OR (qualitative methods) OR interview* OR (semi-structured interviews) or (semi structured interviews) OR (Focus Groups) OR (Grounded Theory)) | 2238532 |
| #4 | #1 AND #2 AND #3 | 88 |
| **CNKI** | | |
| #1 | TKA=(绝经 + 围绝经 + 更年期 + 绝经前期) | 78350 |
| #2 | TKA=(质性研究 + 定性研究) | 196534 |
| #3 | #1 AND #2 | 89 |
|  | **COJ** |  |
| #1 | 主题:(绝经 OR 围绝经 OR 更年期 OR 绝经前期) | 91876 |
| #2 | 主题:(“质性研究” OR “定性研究”) | 57217 |
| #3 | #1 AND #2 | 37 |
|  | **VIP** |  |
| #1 | 题名或关键词=绝经 OR 更年 | 46473 |
| #2 | 题名或关键词=质性 OR 定性 | 356601 |
| #3 | #1 AND #2 | 96 |

\
